# Tintelnotia destructans, the Rare Opportunist of a Behçet's Disease Patient

**DOI:** 10.7759/cureus.81142

**Published:** 2025-03-25

**Authors:** Rita Gano, Diana Cristina Buendia Palacios, Sofia Pinheiro

**Affiliations:** 1 Department of Internal Medicine, Unidade Local de Saúde Santa Maria - Hospital Pulido Valente, Lisbon, PRT; 2 Internal Medicine Service (Functional Unit 2.3), Unidade Local de Saúde São José - Hospital de Santo António dos Capuchos, Lisbon, PRT; 3 Autoimmune Diseases Unit, Unidade Local de Saúde São José, Lisbon, PRT; 4 NOVA Medical School - Faculty of Medical Sciences, Universidade Nova de Lisboa, Lisbon, PRT

**Keywords:** behçet's disease, ocular abscess, opportunistic infection, rare fungus, tintelnotia destructans

## Abstract

Behçet's disease is a systemic vasculitis characterized by recurrent oral and genital ulcers that can have ophthalmologic, cutaneous, neurologic, vascular, and thromboembolic manifestations. Treatment usually involves immunosuppressant medication, which leads to an increased risk of opportunistic infections. Only recently identified, *Tintelnotia* *destructans* is a rare fungus that can cause eye and nail infections in humans, usually refractory to standard antifungal therapy. Ocular infections are most commonly associated with ocular trauma or the use of contact lenses and may cause permanent damage without adequate treatment. We present a case of a 40-year-old man with Behçet's disease, treated with adalimumab, who developed an ocular abscess due to *Tintelnotia destructans*. This clinical case serves the purpose of alerting for an opportunistic infection caused by a newly described and rare microorganism, which is hard to identify.

## Introduction

Behçet's disease (BD) is a rare autoimmune, multisystemic inflammatory disease characterized by recurrent oral and genital ulcers and ocular inflammation [1-5]. As a systemic vasculitis, it can involve the joints, skin, central nervous system, and gastrointestinal tract and affect both arteries and veins in almost any organ. Its etiology is still not completely known, but it is strongly associated with the presence of the HLA-B51 allele [1-5]. Most commonly seen in people of Mediterranean and Eastern origin, there appears to be a relation with an increased prevalence in patients living in areas along the Silk Road, where the HLA-B51 allele positivity is higher, reaching 81% in some areas [6]. This particular geographic spread is thought to be explained by genetic etiology and possible environmental factors [2,3,6]. 

The diagnosis of BD is clinically based, as no specific laboratory test is available. Active systemic disease is usually associated with the elevation of systemic inflammation markers, such as circulating pro-inflammatory cytokine (TNF-a, IL-1b, IL-6, and IL-8) and increased C-reactive protein levels [1,2].

The most common and often earliest sign of disease is recurrent aphthous ulceration, which typically precedes the onset of systemic symptoms. These ulcers can be oral or genital, occurring in 72-94% of cases, while ocular inflammation affects 30-70% of patients [2]. The latter is more severe in men than in women and usually manifests as chronic and relapsing uveitis with associated morbidity. Arthralgia is frequently observed, along with skin lesions such as erythema nodosum, pathergy (hyperreactivity of the skin to minor trauma), and vasculitis involvement, including thrombophlebitis and deep venous thrombosis. For diagnosis, recurrent oral ulceration must be present as well as at least two of the following: recurrent genital ulceration, ocular lesions, skin lesions, or a positive pathergy test [1-3,6].

Treatment options should be tailored to the patient's manifestations. However, management may involve high-dose corticosteroids, immunosuppressant medication, and, depending on the organ involved, surgical intervention. Since ocular lesions can rapidly progress to blindness early in the course of the disease, close monitoring is essential for timely intervention to reduce the severity and frequency of ocular crisis [1-3].

Because BD treatment often leads to immunosuppression, patients have an increased risk of opportunistic infections. Regular screenings for opportunistic and chronic infections, including hepatitis viruses, human immunodeficiency virus (HIV), and tuberculosis, are recommended. Screening should be tailored for regional pathogen prevalence, patient exposure risks, and the specific immunosuppressive medications used [7,8].

*Tintelnotia *is a novel genus of the *Phaeosphaeriaceae *family within the order *Pleosporales *[9-11]. *Tintelnotia destructans* (TD) is a fungus that causes ocular and nail infections in humans. These infections are commonly acquired after minimal trauma and remain localized and superficial; however, dissemination may occur in severely immunosuppressed patients. TD exhibits a variable susceptibility pattern and is often resistant to commonly used antifungal therapies; however, terbinafine has demonstrated a favorable therapeutic response. Currently, no standardized treatment exists, making susceptibility testing crucial for guiding effective therapy [9-11].

## Case presentation

We present the case of a 40-year-old man, with a personal history of previous spontaneous pneumothorax, active smoking, and a two-year diagnosis of BD (HLA-B51 positive), with recurrent arthralgia, oral ulcers, and severe ocular inflammation, specifically posterior uveitis with retinal edema and periphlebitis. He was under follow-up with Internal Medicine and Ophthalmology specialists and was being treated with adalimumab 40 mg weekly and azathioprine 100 mg once daily for disease control. Because of his immunosuppressive therapy, he recently underwent screening for HIV-1 and HIV-2, hepatitis B and C viruses, and tuberculosis, all of which were negative. The patient had no other comorbidities and no family history of autoimmune disease. 

The patient went to the emergency department with a one-day history of decreased visual acuity and a foreign body sensation in his left eye. He was observed by Ophthalmology, who identified a corneal abscess and initiated topical corticosteroid treatment after performing a corneal swab. The swab was cultured for bacteria, fungi, and *Acanthamoeba *and tested with polymerase chain reaction (PCR), but all results remained negative. Laboratory tests revealed no abnormalities, including normal inflammatory markers. 

Given the immunosuppressed status of the patient, an opportunistic infection caused by bacteria (such as *Staphylococcus*, *Streptococcus*, and *Pseudomonas*), fungi (*Fusarium*), and parasites (*Acanthamoeba*) was suspected. His Internal Medicine physician decided to temporarily suspend adalimumab until resolution, a month later. 

**Figure 1:**
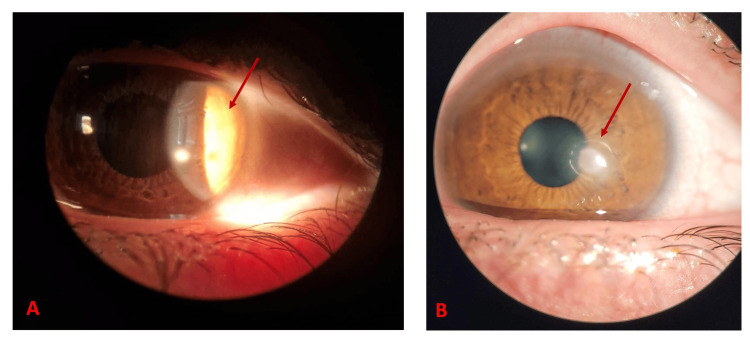
Ocular microscopy of the left eye (A) Slit lamp examination showing edema and hyper-reflective branching hyphae (arrow). (B) Ocular microscopy showing a cotton-like mass and hyper-reflective branching hyphae (arrow).

At follow-up one week later, the patient's symptoms persisted without improvement, and examination revealed that the abscess had enlarged despite corticosteroid treatment. Ocular microscopy findings resembled those typically caused by an *Entamoeba histolytica* infection (Figure 1). Although PCR testing was negative for *E. histolytica*, the patient was empirically started on topical voriconazole and moxifloxacin.

To better characterize the ocular lesion and exclude cerebral involvement, orbital and cranial magnetic resonance imaging (MRI) was performed, including T1 and T2 fluid-attenuated inversion recovery (FLAIR), short tau inversion recovery (STIR) sequences, and gadolinium contrast. The MRI showed only mild asymmetry to the lacrimal glands, with the left gland being slightly larger, a finding consistent with the patient's reported symptoms. No additional abnormalities were detected in the ocular globes, optic nerves, extraocular muscles, or brain.

Two weeks later, TD, a fungus, was identified by PCR and sequence analysis as the causative agent of the abscess. The patient was then started on terbinafine and voriconazole, guided by in vitro susceptibility testing, leading to significant improvement within a few weeks.

## Discussion

The incidence of fungal keratitis in Europe has been increasing, with the majority of cases associated with contact lens use [9]. Until recently, TD was primarily linked to infections acquired after ocular trauma or contact lens wear, which typically remain superficial and localized in immunocompetent patients [12].

In immunocompromised patients, the differential diagnosis of ocular infections should include a broad spectrum of pathogens. These range from bacteria (e.g., *Staphylococcus aureus*, *Pseudomonas aeruginosa*) and mycobacteria to fungi such as *Candida*, *Aspergillus*, and *Fusarium* and emerging fungi like TD. Viral agents (e.g., herpes simplex virus) and parasitic infections such as *Toxoplasma gondii* or *Acanthamoeba *should also be considered, especially in cases unresponsive to standard antimicrobial therapy [8,9,11]. 

Ocular fungal infections are associated with visual impairment and blindness, making the identification of the causative organism essential to determining the most effective treatment. The identification, management, and treatment of TD infections are particularly challenging. In many cases, culture and microscopy can only classify the fungus at the *Phaeosphaeriaceae *family level, requiring PCR and sequence analysis to confirm the genus and species. This makes molecular testing the preferred diagnostic method for identifying the organism, especially when suspicion is high despite negative culture results. 

TD exhibits variable susceptibility to antifungal drugs, which explains its poor response to empiric therapy with standard antifungal agents. Therefore, susceptibility testing is crucial to guide effective treatment. Currently, no standardized therapeutic strategy exists, but terbinafine, an allylamine with antifungal properties, has demonstrated favorable outcomes. While this fungus usually causes localized and superficial infections of the nails and eyes, it may disseminate in immunocompromised patients, leading to severe and potentially life-threatening conditions [9,10].

Ocular disease occurs in 30-70% of BD patients who develop ocular inflammation. The progressive ischemic damage seen in BD can rapidly lead to blindness early in the disease course. Management includes anti-inflammatory and immune-modulating drugs, the latter of which can induce immunosuppression [1-3].

Patients with autoimmune inflammatory diseases are at increased risk for opportunistic infections due to immune system dysregulation and immunosuppressive treatments. Therefore, they should undergo regular screening for chronic and opportunistic infections, including tuberculosis, hepatitis B and C viruses, *Pneumocystis jirovecii*, and other pathogens, based on exposure risk and treatment regimen [7,8].

In this case, the patient was on adalimumab and azathioprine, which increased the risk of opportunistic infections. However, he was closely monitored by Internal Medicine and Ophthalmology specialists for both disease progression and potential infections. When the ocular infection was diagnosed, accurate identification of the causative pathogen became a priority, as it would guide appropriate treatment and prevent dissemination, which could lead to serious complications.

Microscopy and culture failed to identify the pathogen, consistent with reports in the literature [9]. TD was only identified by PCR and sequence analysis two weeks after the initial presentation, and during that time, the patient remained on empiric therapy without improvement. However, after switching to targeted antifungal therapy based on susceptibility testing, the patient showed significant clinical improvement, with infection resolution after a few weeks of terbinafine and voriconazole. Since the patient had no history of ocular trauma or contact lens wear, immunosuppression was considered the main predisposing factor for this opportunistic fungal infection.

## Conclusions

This case highlights the challenges of diagnosing and managing infections caused by TD, a rare and newly described opportunistic fungus. In an immunosuppressed patient with BD, the delayed identification of TD led to an extended period of empiric therapy with limited effectiveness, underscoring the critical need for early and accurate microbiological diagnosis. Given the variable antifungal susceptibility of TD, susceptibility-guided therapy was essential for treatment success, resulting in clinical resolution following the initiation of terbinafine and voriconazole.

By reporting this case, we aim to raise awareness of TD as an emerging pathogen in immunocompromised individuals and to stress the importance of maintaining a high index of suspicion for rare fungal infections to enable rapid diagnosis and susceptibility-guided treatment. In this case, the patient responded favorably to targeted antifungal therapy, with complete resolution of the abscess, restoration of visual acuity, and no residual deficits or sequelae. Early recognition and appropriate management were key to achieving a successful outcome.
